# Correction to mother's functional difficulty is affecting the child functioning: Findings from a nationally representative MICS 2019 cross‐sectional survey in Bangladesh

**DOI:** 10.1002/hsr2.1072

**Published:** 2023-01-18

Anjum, A, Ahammed, T, Hasan, MM, Chowdhury, MAB, Uddin, MJ. Mother's functional difficulty is affecting the child functioning: findings from a nationally representative MICS 2019 cross‐sectional survey in Bangladesh. *Health Sci Rep*. 2022;6:e1023. doi:10.1002/hsr2.1023

There was an incorrect value in Table 1 for 5−17 year children in the Division Barisal. The weighted percent should be 21 instead of 2.1.

We apologize for this error.

